# Rationale of combination of anti-PD-1/PD-L1 antibody therapy and radiotherapy for cancer treatment

**DOI:** 10.1007/s10147-020-01666-1

**Published:** 2020-04-03

**Authors:** Hiro Sato, Noriyuki Okonogi, Takashi Nakano

**Affiliations:** 1 Department of Radiation Oncology, Graduate School of Medicine, Gunma University, Maebashi, Gunma 371-8511 Japan; 2 National Institute of Radiological Sciences, National Institute for Quantum and Radiological Science and Technology, Inage, Chiba 263-8555 Japan

**Keywords:** Radiotherapy, Immunogenic cell death, Immune checkpoint inhibitors, PD-1, PD-L1

## Abstract

Significant technological advances in radiotherapy have been made in the past few decades. High-precision radiotherapy has recently become popular and is contributing to improvements in the local control of the irradiated target lesions and the reduction of adverse effects. Accordingly, for long-term survival, the importance of systemic cancer control, including at non-irradiated sites, is growing. Toward this challenge, the treatment methods in which anti-PD-1/PD-L1 antibodies that exert systemic effects by restoring anti-tumour immunity are combined with radiotherapy has attracted attention in recent years. Previous studies have reported the activation of anti-tumour immunity by radiotherapy, which simultaneously elevates PD-L1 expression, suggesting a potential for combination therapy. Radiotherapy induces so-called ‘immunogenic cell death’, which involves cell surface translocation of calreticulin and extracellular release of high-mobility group protein box 1 (HMGB-1) and adenosine-5′-triphosphate (ATP). Furthermore, radiotherapy causes immune activation via MHC class I upregulation and cGAS–STING pathway. In contrast, induction of immunosuppressive lymphocytes and the release of immunosuppressive cytokines and chemokines by radiotherapy contribute to immunosuppressive reactions. In this article, we review immune responses induced by radiotherapy as well as previous reports to support the rationale of combination of radiotherapy and anti-PD-1/PD-L1 antibodies. A number of preclinical and clinical studies have shown the efficacy of radiotherapy combined with immune checkpoint inhibition, hence combination therapy is considered to be an important future strategy for cancer treatment.

## Introduction

Radiotherapy (RT) is a major form of cancer therapy and is used to treat many types of cancer, regardless of clinical stage. The last few decades have seen remarkable advances in RT that have enabled the use of higher local radiation dose with fewer fractions while minimising the dose to surrounded non-target tissue [1]. Several RT modalities are widely prevalent in clinical practice today, including intensity-modulated radiation therapy (IMRT), stereotactic body radiotherapy (SBRT) and stereotactic radiosurgery (SRS). In addition, particle therapy (proton or carbon-ion radiotherapy) has been covered by insurance in Japan since 2016, although its use is limited to certain types of cancer. While these technical advances have contributed to improvements in the local control of irradiated tumours, control of systemic disease is required for long-term survival of patients.

Anti-PD-1/PD-L1 antibodies blocks the immune checkpoint pathway and restores the activity of activated T cells against tumours [2, 3]. PD-1 blockade has spectacular results in patients even with an advanced stage cancer [4–12]; however, the impressive responders are around only 10% of the patients and 20–40% of patients still exhibit progressive disease. For this reason, methods of using anti-PD-1/PD-L1 antibodies in combination with conventional cancer treatments are under active exploration. Among them, RT is a promising candidate because preclinical and clinical evidences have demonstrated that RT elicits immune responses, including both stimulation and suppression as well as DNA damage. Therefore, escape from immune suppression after RT enables appropriate systemic anti-tumour immune activation. RT-induced systemic immune activation has potential that leads to shrinking of distant lesions outside the irradiated field, i.e. an abscopal effect. In the past, abscopal effect was a very rare phenomenon. However, recent several clinical reports have shown that the combination of RT and anti-PD-1/PD-L1 antibodies can induce the abscopal effect, suggesting that the combined therapy is promising because of complementary and synergistic anti-tumour effects. The present article summarises the immunological rationale for the combination of RT with anti-PD-1/PD-L1 antibodies and reviews the emerging preclinical and clinical evidence for this strategy.

## Preclinical evidences on the immune responses upon irradiation

### Immune activation by irradiation

Numerous preclinical studies to date have revealed immune activation by irradiation. Irradiation activates host immunity by triggering immunogenic cell death (ICD), which is characterised by the release of damage-associated molecular patterns (DAMPs) that activate dendritic cells (DCs), presenting tumour antigens and priming antigen-specific T cells in a dose-dependent manner [13]. ICD consists of: (1) cell surface translocation of calreticulin (CRT); (2) extracellular release of high-mobility group protein box 1 (HMGB-1); and (3) extracellular release of adenosine-5′-triphosphate (ATP) [14]. CRT is an endoplasmic reticulum (ER)-resident chaperone that promotes phagocytosis of irradiated tumour cells by DCs when it is present on tumour cell surfaces [15]. HMGB1 is a nuclear DNA-binding protein that acts as toll-like receptor 4 (TLR4) agonist and activates DCs via both TLR4 and the receptor for advanced glycation end products [16, 17]. It has been shown that HMGB1-dependent TLR4/MyD88/TRIF signalling leads to T cell activation [18, 19]. Gameiro et al. analysed ICD by irradiation and found that CRT, HMGB1 and ATP were induced after cell line gamma ray irradiation [20]. Furthermore, they found that CRT expression was also induced on the surface of irradiated tumour cells after RT of nude mice implanted with human tumour cell lines. More recently, research on ICD using particle beams is growing. Several groups including us have reported that the release of HMGB1 and the expression of CRT after particle therapy are at least comparable to conventional X-rays [21–23]. ATP is an intercellular signalling factor that attracts DCs to tumours by binding to their P2X7 purinergic receptors [13]. Activated DCs secrete IL-1β, leading to priming of interferon-γ-producing CD8+T cells [24]. Thus, irradiation-induced immune activation via ICD, which leads to DCs and antigen-specific T cell activation, is supported by lots of preclinical data.

In addition to ICD, interferons (IFNs) are also important for immune activation induced by RT. The type I IFN (α and β) pathway is upregulated via the cyclic GMP–AMP (cGAMP) synthase (cGAS)–stimulator of interferon genes (STING) pathway after irradiation [25]. cGAS recognises cytoplasmic DNA and catalyses the synthesis of cGAMP, which functions as a secondary messenger that binds to and activates the adaptor protein STING. Activation of the cGAS–STING pathway induces type I IFNs production through IRF3/NFκB-dependent transcriptional activation [26, 27]. Irradiation-induced type I IFNs enhance cross-priming of DCs, which is required for the tumour-shrinking effect of RT [28]. The recognition of cytoplasmic DNA was originally discovered as a fundamental mechanism of the innate immune system for sensing the presence of microbial pathogens [29, 30]. Importantly, such cytoplasmic DNA is generated during mitosis in cancer cells following DNA damage by irradiation, suggesting that it acts as a kind of DAMPs. Indeed, the combination of intramuscular delivery of cGAMP and anti-PD-L1 antibody inhibits tumour growth and prolongs mouse survival more than either treatment alone [31]. Type II IFNs (e.g. IFNγ) also play a crucial role in tumour elimination by RT, since intratumor IFNγ levels are significantly increased by RT and IFNγ knock-out mice fail to control tumour growth by RT [32].

MHC class I expression is another factor in immune activation after RT. MHC class I molecules present intracellular antigenic peptides that are generated by proteasomes and translocated into ER by the transporter associated with antigen processing. MHC class I–peptide complexes then move to the cell surface to be recognised by CD8-positive T cells. Importantly, MHC class I expression and antigen presentation by cancer cells upregulate after irradiation. Reits et al. showed that gamma ray irradiation increases intracellular peptide and protein synthesis via mTOR activation, resulting in a dose-dependent increase in MHC class I expression [33]. Tumour antigen presented by MHC class I as well as the release of tumour antigens from dying cells induces a tumour-specific T-lymphocyte response. Irradiation also activates NK cell-mediated cytotoxicity via activation of natural killer receptor G2D (NKG2D) ligands, which are upregulated by ATM [34–36]. Thus, the immunogenic release of DAMPs and IFNs and elevated antigen presentation by upregulation of MHC class I molecules contribute to the enhanced susceptibility of irradiated tumours to immune responses.

**Fig. 1:**
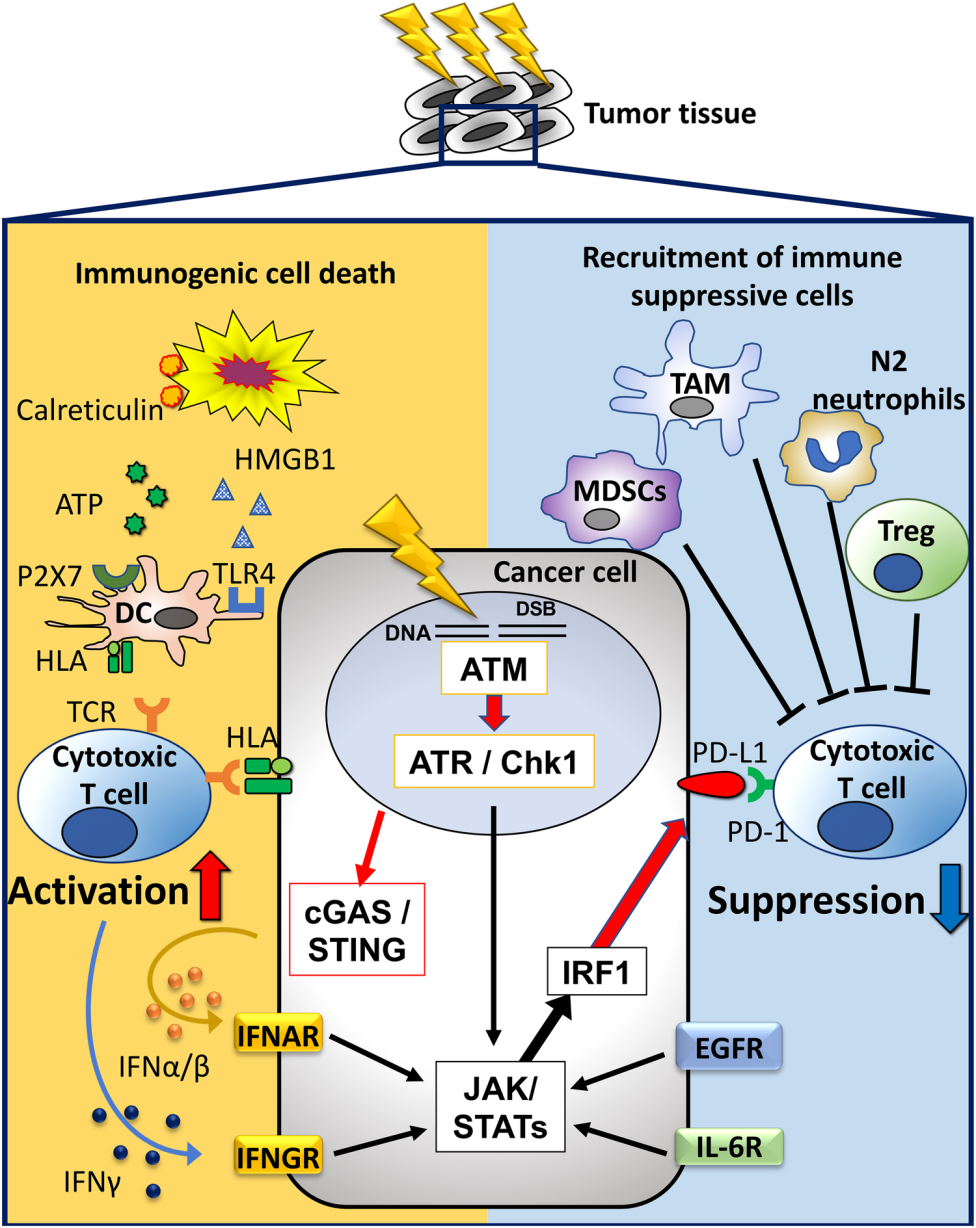
Immune responses induced by radiotherapy. Radiotherapy induces both immune stimulative and immune suppressive responses. Left side: radiotherapy (RT) causes immunogenic cell death (ICD), which releases HMGB1 and ATP, and expresses calreticulin. ICD recruits and activates DC in the tumour microenvironment, resulting in cytotoxic T cell activation. DNA double-strand breaks generate micronuclei, which activate the cGAS–STING pathway, which then upregulates the release of type I IFN. Right side: RT recruits immune suppressors, such as M2 tumour-associated macrophages (TAMs), myeloid-derived suppressor cells (MDSCs) and regulatory T cells (Tregs). As a mechanism of PD-L1 expression induced by RT the following four pathways have been reported: (1) IFN/IL-6, (2) EGFR, (3) DNA damage and repair signal, and (4) cGAS–STING. In any of the routes, finally PD-L1 expression is induced via the STAT/IRF pathway

Taken together, these evidences strongly suggest that RT primes the tumour microenvironment to be sensitive to treatment with an immune checkpoint inhibitor. Figure 1 (left side) summarises the major response of immune stimulation after irradiation.

### Immune suppression by irradiation

In addition to immune activation, RT also cause immunosuppressive effects in the tumour microenvironment due to the attraction of immunosuppressive cells such as M2 tumour-associated macrophages (TAMs), myeloid-derived suppressor cells (MDSCs), regulatory T cells (Tregs) and N2 neutrophils as well as because of the release of immunosuppressive cytokines (TGF-β and IL-10) and chemokines [37].

Furthermore, a number of preclinical studies have reported irradiation-induced PD-L1 expression [38–40]. The IFN-dependent pathway, a canonical pathway for PD-L1 expression, also upregulates PD-L1 expression after irradiation. Although both type I and II IFNs can upregulate PD-L1, IFNγ induction of PD-L1 is stronger and more persistent via JAK–STAT–IRF pathway [41–43]. Dovedi et al. analysed PD-L1 upregulation by X-ray RT using tumour-bearing mice [38]. They found that PD-L1 upregulated with a peak 3 days after the last prescription of fractionated RT, which requires IFNγ production by CD8-positive T cells. Conversely, they also reported that in vitro single fractions of 2.5–10 Gy for the same murine cancer cell lines did not effectively induce PD-L1 expression, suggesting the importance of IFNγ in PD-L1 upregulation after X-ray irradiation. In addition, because type I IFN induces PD-L1 expression, cGAS–STING pathway is also an important upstream signal for PD-L1 expression. Recently, type I IFN induction via cGAS–STING pathway was shown to be induced after 24 Gy in three fractions of murine mammary tumour cell-bearing mice [44]. The IL-6/JAK/STAT pathway is also involved in PD-L1 upregulation in human oesophageal cancer cells after irradiation [39]. Additionally, EGFR signalling after irradiation contributes to PD-L1 expression via the IL-6/JAK/STAT3 pathway [45, 46]. This PD-L1 expression was mediated by irradiation-dependent phosphorylation of EGFR (Y1173) and JAK2 (Y1007, Y1008) and was suppressed by inhibiting JAK2 phosphorylation, supporting the importance of EGFR–JAK signalling in PD-L1 expression after irradiation.

Recent preclinical studies have revealed that the response to DNA damages (e.g. DNA double-strand breaks, DNA single-strand breaks and base damage) upregulates PD-L1 expression in cancer cells via ATM/ATR/Chk1 kinase activation [47–49]. These data were supported by experiments with mouse tumour models using a specific ATR inhibitor where RT-induced PD-L1 upregulation was significantly suppressed, resulting in the attenuation of RT-induced CD8-positive T cell exhaustion and cancer cells sensitised to the cytotoxicity of CD8-positive T cells [50, 51]. These studies suggest that the ATR/Chk1 activity followed by the activation of STAT–IRF pathway, rather than DNA damage per se, is a central factor that affects PD-L1 upregulation after irradiation.

As described above, these evidences indicate that RT induces immunosuppression as well as immune activation. However, anti-PD-1/PD-L1 antibodies have potential to relieve this immunosuppression, which makes sense as a combination therapy after RT. Figure 1 (right side) illustrates the major immune suppressive response, including PD-L1 upregulation, after irradiation.

## Preclinical evidences on combined therapy

As discussed in the previous section, the response of the immune system to irradiation includes both activated and suppressive effects. An important suppressive response is the induction of PD-L1 expression, which may be overcome by combination with anti-PD-1/PD-L1 antibodies. This section presents preclinical data indicating the efficacy of combined therapy.

In 2013, Zeng et al. reported that combining anti-PD-1 antibody and stereotactic RT improves survival in mice with intracranial gliomas [52]. Dovedi et al. found that concurrent, but not sequential administration of anti-PD-1/PD-L1 antibodies with fractionated RT, which is the regimen adopted in conventional RT, is required to achieve long-term tumour control [38]. High-dose RT (12–20 Gy in a single fraction) combined with anti-PD-1/PD-L1 antibodies for tumour-bearing mice induced abscopal effect that suppressed the growth of an unirradiated tumour re-challenged on the opposite flank, suggesting the induction of persistent systemic anti-tumour immune response by the combined therapy in tumour-bearing mouse model studies [53, 54]. As the background of abscopal effect, stereotactic RT elicited several immune responses, including upregulation of antigen cross-presentation in draining lymph nodes by tumour-specific antigen–MHC complexes and increase in tumour T cell infiltration [55]. RT also upregulated the expressions of CD137 and PD-1 in CD8-positive tumour-infiltrated lymphocytes in tumour-bearing mice and the abscopal effect was enhanced by triple combination of RT with anti-PD-1 and anti-CD137 antibodies [56]. Furthermore, RT followed by anti-PD-1 antibody significantly increased the CD8+/Treg ratio and PD-L1 expression in tumour cells, resulting in tumour growth suppression and prolonged survival in a mouse NSCLC model [57]. Another tumour-transplanted mouse analysis of combined RT with anti-PD-L1 antibody treatment demonstrated that the number of MDSCs and Tregs in the tumour decreased, whereas the number of CD8-positive T cells increased, suggesting that the control of immunosuppression by combined treatment can contribute to the inhibition of tumour growth [58]. Furthermore, recently, Takahashi et al. reported that carbon-ion radiotherapy combined with anti-PD-L1 antibody and anti-CTLA-4 antibody delayed tumour growth not only in the irradiated tumours but also in the unirradiated tumours [59]. It is important to note that they used both anti-CTLA-4 as well as anti-PD-L1 antibodies. They reported that, notably, 64% of mice in the combined treatment group showed complete response of unirradiated tumours. Thus, over the last decade, preclinical data have been accumulated to demonstrate the efficacy of anti-PD-1/PD-L1 antibodies combined with RT. These evidences provide the basis for current clinical trials of combined therapy.

## Clinical and translational evidences on immune response by radiotherapy

In addition to preclinical data, a number of clinical studies now reveal an immune response by RT. In this section, we present clinical and translational evidences supporting preclinical data.

Several clinical reports have shown the induction of ICD by RT. Suzuki and Mimura et al. analysed the ICD induction by chemoradiotherapy in patients with oesophageal squamous cell carcinoma [60]. They reported that preoperative chemoradiotherapy upregulates HMGB1 both within the tumour microenvironment and the serum of patients. In addition, serum HMGB1 was significantly higher in patients who showed antigen-specific T cell responses compared with non-responsive patients, suggesting that the HMGB1 produced by chemoradiation plays an important role in inducing tumour antigen-specific T cells. Importantly, the patient group that strongly expressed HMGB1 exhibited significantly better overall survival (OS) than the low-expressing group, regardless of whether chemoradiotherapy was used or not, indicating that HMGB1 independently contributes to improved survival. Singh et al. also reported ICD after RT [61]. They showed that SBRT increases tumour cell surface expression of CRT in patient with renal cell carcinoma. Thus, because (chemo)radiotherapy elicits ICD, it can contribute for systemic immune stimulative condition.

Many types of cancers exhibit downregulation of MHC class I to escape the immune response, since then impeded their detection by T cells and contribute to the immunosuppressive microenvironment. Indeed, low expression of human leukocyte antigen (HLA), human MHC, correlates with poor clinical outcome [62]. Consistent with in vitro and in vivo data, however, we reported that HLA class I is upregulated by hyperthermochemoradiotherapy in patients with rectal cancer [63].

The PD-1/PD-L1 axis is one of the key factors in cancer immune escape induced by RT, because clinical reports have shown that high PD-L1 expression by tumours is associated with poor prognosis [39, 64, 65]. To date, upregulation of PD-L1 expression has been reported in patients with rectal cancer, non-small cell lung cancer, oesophageal cancer and soft tissue sarcoma (STS) who have undergone RT with or without chemotherapy as preoperative treatment. Neoadjuvant chemoradiotherapy for rectal and oesophageal cancer and preoperative conventional X-ray RT for STS-induced PD-L1 expression in tumour cells have been reported [66–70]. More recently, PD-L1 upregulation induced by carbon-ion radiotherapy in patient with uterine cervical adeno/adenosquamous carcinoma was reported [49]. On the other hand, a few other studies have reported that PD-L1 expression in tumour cells did not show a significant change even after neoadjuvant chemoradiotherapy for the treatment of rectal cancer [71, 72]. Further, conversely, a report has stated that PD-L1 expression was increased only in 11%, while decreased in 45% of patients with NSCLC after preoperative chemoradiotherapy [73]. Importantly, their study indicates that PD-L1 increased patients had poor survival compared to PD-L1 decreased or unchanged group. These data imply that PD-L1 upregulation induced by RT may contribute to immune evasion, which leads to poor outcome. But, on the other hand, PD-L1 expression is also considered as one of the predictive markers for anti-PD-1/PD-L1 antibodies therapy responsiveness. Early clinical trials of PD-1/PD-L1 blockade suggested a prolonged survival of patients with PD-L1 positive tumours [8, 74, 75]. A review of PD-1/PD-L1 blockade in 17 clinical studies reported that the objective response rate (ORR) in patients with PD-L1-positive tumours was 48% compared with 15% in patients with PD-L1-negative tumours [76]. Together, changes in the immune environment following RT in patients promote immune activation and also induce PD-L1 expression, creating a situation suitable for the combined use of anti-PD-1/PD-L1 antibodies.

## Clinical and translational evidences on combined therapy

As discussed above, preclinical and clinical studies have described that irradiation promotes both immune activation and immunosuppression. In other words, RT functions as both the accelerator and brake of the antitumor immune system. Therefore, theoretically, if the brake is released by combining with the anti-PD-1/PD-L1 antibody, more effective elimination of the tumour by the immune system can be expected.

The results of a phase III clinical trial on patients with locally advanced, unresectable NSCLC (named the Pacific trial) have had a major impact on the field of clinical oncology. In this study, progression-free survival (PFS) was significantly prolonged by prescribing durvalumab as a consolidation therapy within 1–42 days after concurrent chemoradiotherapy as compared with placebo [77]. In addition, the subgroup analysis showed that OS was also significantly prolonged in patients treated with durvalumab within 1–14 days after completion of chemoradiotherapy [78]. With regard to toxicity, importantly, the rate of grade 3 or 4 adverse events of any cause was comparable, suggesting the safety of durvalumab following chemoradiotherapy. Based on this trial, durvalumab following chemoradiotherapy has been approved for the treatment of NSCLC by Food and Drug Administration as well as by other countries. Unfortunately, this is the only reported combination therapy phase III trial so far, although several early-phase trials have also supported the efficacy of combination treatment.

Similar to Pacific trial, early clinical trials and retrospective analyses have also reported the efficacy of sequential combination. A phase I study of multisite SBRT combined with sequential pembrolizumab in patients with metastatic solid disease was conducted. The overall ORR was 13.2% (CR: 1.5%, PR: 11.8%) with a median OS of 9.6 months. In addition, the abscopal effect was observed in 26.9% of cases [79]. In this study, grade 3 toxicities (pneumonitis, colitis and hepatic toxicity) were observed in 8.2% of cases. Interestingly, patients treated with anti-PD-1/PD-L1 antibody, those with a history of previous RT had greater PFS, OS and response rate than those without [80–82]. Although these were not clinical trials for combined therapy, these data support the possibility that immune activation by RT may further enhance the effect of anti-PD-1/PD-L1 antibody. In contrast, several studies have shown the efficacy of concurrent strategy. A retrospective analysis of patients with metastatic melanoma reported that the response rate of patients concurrently treated with SRS and anti-PD-1 antibody was 64% compared with that of 44% for sequentially treated patients [83]. Although the difference was not significant, this data may support the preclinical evidences that the immune responses induced by RT are temporary and theoretically concurrent administration may contribute to a better response rate. Another phase I/II study demonstrated the feasibility of concurrent treatment of palliative local RT and durvalumab [84]. Although palliative RT doses, i.e. the number of fractions and patients’ background varied, 60% objective response (20% of CR and 40% of PR) was observed in the irradiated field without grade 3 or higher adverse events. In this phase I/II study, 71% of tumour growth suppression of non-irradiated tumour, but no shrinkage (no abscopal effect), was observed. Further, concurrent combination improved response rate and abscopal effects in patient with metastatic melanoma [85, 86]. In this manner, a number of other retrospective case series support the notion of a combination strategy of RT and anti-PD-1/PD-L1 antibody therapy, including both concurrent and sequential use [87]. Taken together, although the precise mechanisms contributing to better prognosis are still unclear, RT may sustainably maintain the host immunity in favourable immune environments with anti-PD-1/PD-L1 antibody treatment as well as temporary immune response.

## Perspectives

The combination of RT and anti-PD-1/PD-L1 antibody is a promising strategy supported by a number of preclinical and clinical evidences. In the present day, nearly 100 clinical trials of combined therapy are ongoing [37]. However, there are many points that have not yet been clarified, such as the optimal combination timing and dose fraction.

In point of the combination timing, according to preclinical data, ICD-induced tumour-specific T cell responses and PD-L1 upregulation are transient, so theoretically, concurrent combination might be better [47, 60]. On this subject, the clinical trial of chemoradiotherapy and durvalumab concurrent combination (so-called Pacific2 trial) for unresectable NSCLC is expected to give a hint whether concurrent combination or sequential combination is a better combination strategy [88]. In addition, there have been some reports that anti-PD-1/PD-L1 antibody was particularly effective in patients with a history of RT. Therefore, in the future, to evaluate the precise mechanisms of combined therapy, analysis of long-term immunity induction by RT using clinical samples is also required.

For optimal dose fractions, there are reports that support the efficacy of both single fraction [89] and fractionated regimens [90, 91] in the induction of abscopal effects. With respect to the optimal dose for inducing immune responses, it has been shown that a single high-dose irradiation (20 Gy in a single fraction) inhibits type I IFN production via the cGAS–STING pathway, resulting in the subsequent reduction in immunogenicity [44]. These results indicate that although a high dose is beneficial for the treatment as they induce DNA damage, the optimal dose to promote immune activation is different. Thus, further studies will be necessary to clarify the optimal combination approach to achieve activation of effective anti-tumour immunity and clinical benefit [92].

## Conclusion

Immune responses induced by RT are becoming apparent in both preclinical and clinical levels. Because of these immune responses, after RT, the tumour microenvironment became appropriate to exert the effects of anti-PD-1/PD-L1 antibodies not only in the local tumour microenvironment but also systemically. On the contrary, there are still challenges for clinical details, e.g. the high response rate of anti-PD-1/PD-L1 antibody in patients even after a long interval from RT history. It is also necessary to establish a method for selecting patients who would benefit from combination therapy. While there are still many questions to be addressed to further improve the combination of RT and anti-PD-1/PD-L1 antibodies treatment, it is clear that this new modality has opened up a new era in clinical oncology.
